# Impact of cow milk production on cow–calf performance in the Nebraska Sandhills

**DOI:** 10.1093/tas/txaa123

**Published:** 2020-12-22

**Authors:** Tasha M King, Jacki A Musgrave, Richard N Funston, J Travis Mulliniks

**Affiliations:** 1 Department of Animal Science, University of Nebraska–Lincoln, Lincoln, NE; 2 West Central Research and Extension Center, University of Nebraska, North Platte, NE

## INTRODUCTION

Livestock producers have tended to select for increased output traits like milk production and growth to increase productivity. Even with the increased selection for greater calf growth potential, some regions in the United States have seen a plateau in calf body weight (BW) at weaning (Lalman et al., 2019). When focusing on reaching maximum potential of these output traits, it is important to consider the multitude of variables that affect a production system. With increased milk production, nutrient requirements for cows become increased (Ferrell and Jenkins, 1984; Montaño-Bermudez et al., 1990), which may not be met if range and forage availability for grazing is already limited at meeting lactation demands.

Historically, weaning weight and milk production have been associated with a positive relationship with greater milk production resulting in heavier calves at weaning (Clutter and Nielsen, 1987; Abdelsamei et al., 2005). In contrast, others have only observed the benefit of increased milk production improving calf performance within the first 60 d after birth (Clutter and Nielsen, 1987; Ansotegui et al, 1991; Edwards et al., 2017). Gleddie and Berg (1968) reported the correlation between average daily gain (ADG) of calves and milk yield estimates increased between the first and second month and continued to decrease thereafter as the forage consumption increased. The reliance on milk for dietary energy can result in increased calf BW at peak lactation (Edwards et al., 2017), but benefits of increased milk production may decrease as stage of lactation increases. Our hypothesis was that increasing milk production would negatively affect cow reproductive performance while having no effect on calf performance. Therefore, the objective of this study was to determine the impact milk production has on subsequent cow reproductive performance and calf performance throughout the preweaning and postweaning phases.

## MATERIALS AND METHODS

All animal care and management procedures were reviewed and approved by the University of Nebraska Institutional Care and Animal Use Committee (IACUC approval number 1474).

Data were collected between the years 2000 to 2018 from the March calving herd at the University of Nebraska Gudmundsen Sandhills Laboratory (Whitman, NE). Cows (*n* = 348) utilized were Husker Reds (5/8 Red Angus and 3/8 Simmental) and were 2 to 11 yr of age. In year 2000 and 2015 to 2018, cows were assigned to one of two grazing treatments: meadow or range. From years 2001 to 2014, all cows were grazed on upland range.

### Animal Measurements

Cow BW and body condition score (BCS; Wagner et al., 1988) were recorded in June, July, September, November, and January. Milk production was estimated using the weigh-suckle-weigh method in June, July, September, and November. Calves were separated from cows by 1000 h and allowed to suckle at 1700 h before being separated again. Calf BW were taken at 0700 h the following morning at which time cows and calves were paired up, allowing calves to suckle. Upon completion of suckling period (not exceeding 30 min), calves were weighed again. Difference in calf BW was calculated and used to extrapolate for milk production over 24 h. Ultrasound was used each September for detection of pregnancy to determine reproductive performance of cows.

In each year, calf BW were recorded at birth (March/April), June, July, September, and November. Calf BW at weaning was adjusted to a 205-d age constant BW without adjusting for age of dam and sex of calf. In years 2009, 2011 to 2012, and 2015 to 2017, a subset of calves (*n* = 87) were held in a drylot on ad libitum hay for 2 wk postweaning and then shipped to a feedlot at the West Central Research and Extension Center (North Platte, NE) to be finished. Upon arrival at the feedlot, all steer calves were implanted with 14 mg of estradiol benzoate and 100 mg of trenbolone acetate (Synovex Choice, Zoetis, Parsippany, NJ). Adapted over a 21-d period, calves were finished on a diet containing 48% dry rolled corn, 40% corn gluten feed, 7% grass hay, and 5% supplement. Calves were slaughtered at a commercial abattoir (Tyson Fresh Meats, Lexington, NE) when estimated to visually have 1.27-cm backfat (BF) and carcass data were collected 24 h post-slaughter.

### Statistical Analysis

Data collected throughout the lactation period were averaged and used as variables in the models. The milk production model included cow age, cow BW, and BCS as fixed effects. Cow reproductive performance and calf performance models included milk production as a fixed effect with the addition of cow age, cow BW, and BCS as random effects to account for their influence on milk production. Year and cow served as random effects in all models. Significance level was set at an α ≤ 0.05. All data were analyzed using R (R Core Team, 2017).

## RESULTS AND DISCUSSION

### Cow Performance

Average milk production throughout the lactation period was positively influenced by cow BW and cow age (*P* < 0.001; Table 1). Every increase in kilogram of BW resulted in a 0.012-kg increase in milk production, which is greater than reported by McMorris and Wilton (1986) of 0.003-kg increase in milk production per kg of cow BW. In addition to cow BW, cow age has been shown to affect milk production within the first three lactations and plateau after that (Clutter and Nielsen, 1987). However, milk production has also been shown to decrease after 8 yr of age (Boggs et al., 1980). The current study observed an increase of 0.203 kg in milk production for every additional increase in cow age. Body condition score decreased (*P* < 0.001; Table 1) as milk production increased resulting in a decrease of 0.979 kg of milk production per increase of BCS. A similar response was observed by Boggs et al. (1980) who reported cows with increased milk production had lower BCS in the first 4 mo postpartum.

**Table 1.** Impact of cow demographics on average milk production (kg)^1^

	Coefficient	SE
Intercept	4.630	1.386
Cow BW, kg	0.012	0.002
Cow age	0.203	0.069
Cow BCS	−0.979	0.265
Fit statistics		
*N*	330	
$R_{m}^{2}$^2^	0.310	
$R_{c}^{2}$^2^	0.609	
σ _s_^3^	0.833	
σ _y_^4^	0.637	
σ _Ɛ_^5^	1.198	

^1^All coefficients are *P* < 0.05.

^2^ $R_{m}^{2}$ = *R*^2^ marginal; $R_{c}^{2}$ = *R*^2^ conditional.

^3^Square root of the estimated variance associated with random effects of cow.

^4^Square root of the estimated variance associated with random effects of year.

^5^Square root of the estimated variance associated with residual error.

Milk production did not influence reproductive performance in the current study. Cow pregnancy rate and subsequent calving date were not affected (*P* ≥ 0.80) by milk production. In agreement, no influence of milk production on gestation length was observed by McMorris and Wilton (1986). However, Edwards et al. (2017) observed a decrease in pregnancy rate in cows producing the greatest milk production. These results are in contrast with our current study; however, the average milk production, throughout the data collection period (June to November), of 6.22 ± 1.85 kg/d may have not provided enough variance to detect a difference.

### Preweaning Calf Performance

Increases in adjusted 205-d calf weaning BW and preweaning ADG were observed due to milk production. Preweaning ADG increased (*P* < 0.01; Table 2) by 0.035 kg per kg of increased milk production. Beal et al. (1990) identified a correlation between individual milk production and preweaning calf growth, supporting the increase that was observed in preweaning ADG in the current study. This was reflected in adjusted 205-d calf weaning BW increase (*P* < 0.01; Table 2) of 6.6 kg of calf BW per kg increase of milk production, which is slightly lower than the gain of 7.89 kg reported by Mulliniks et al. (2020). In contrast, Edwards et al. (2017) reported no differences in calf BW after ~day 58 postpartum, which may be due to differences in forage quality consumed by the suckling calves. After 60 d of age, calf preweaning ADG has been shown not to be different between dams with differing milk production levels (Clutter and Nielsen, 1987; Ansotegui et al., 1991; Edwards et al., 2017). However, in agreement with the current study, Clutter and Nielsen (1987) reported increased dam milk production resulted in greater calf BW at 205-d adjusted weaning.

**Table 2.** Milk production influences on preweaning performance of calves^1^

	Preweaning ADG		Adjusted 205-d WW	
	Coefficient	SE	Coefficient	SE
Intercept	0.694	0.026	155.2	6.175
Milk production	0.035	0.003	6.558	0.670
Fit statistics				
*N*	330		330	
$R_{m}^{2}$^2^	0.113		0.216	
$R_{c}^{2}$^2^	0.616		0.617	
σ _s_^3^	0.045		8.490	
σ _b_^4^	0.019		2.271	
σ _a_^5^	0.054		9.263	
σ _y_^6^	0.056		9.356	
σ _Ɛ_^7^	0.080		15.48	

^1^All coefficients are *P* < 0.05.

^2^ $R_{m}^{2}$ = *R*^2^ marginal; $R_{c}^{2}$ = *R*^2^ conditional.

^3^Square root of the estimated variance associated with random effects of cow.

^4^Square root of the estimated variance associated with random effects of cow body condition score.

^5^Square root of the estimated variance associated with random effects of cow age.

^6^Square root of the estimated variance associated with random effects of year.

^7^Square root of the estimated variance associated with residual error.

### Postweaning Performance

Final live calf BW after the finishing phase was increased (*P* = 0.04; Table 3) by 7.9 kg per every additional kg of milk production. In addition, HCW reflected this increase (*P* = 0.04) with an additional 5.0 kg of HCW per kg of average milk production. These increases could be due to the impact of increased milk production on calf weaning BW resulting in heavier calves entering the feedlot. However, feedlot ADG was not affected (*P* = 0.80) by dam milk production. In agreement with the current study, Abdelsamai et al. (2005) reported similar feedlot ADG, but the greater weaning BW calves consumed more milk and had decreased days on feed. Carcass characteristics including ribeye area, BF, marbling score, and final yield grade were not affected (*P* > 0.05) by dam milk production.

**Table 3.** Milk production influences on postweaning performance of calves^1^

	Final live BW		Hot carcass weight	
	Coefficient	SE	Coefficient	SE
Intercept	549.5	29.45	346.2	18.56
Milk production	7.949	3.822	5.008	2.408
Fit statistics				
*N*	87		87	
$R_{m}^{2}$^2^	0.040		0.040	
$R_{c}^{2}$^2^	0.321		0.325	
σ _s_^3^	0.000		0.000	
σ _a_^4^	10.25		6.456	
σ _y_^5^	37.09		23.368	
σ _Ɛ_^6^	56.69		37.61	

^1^All coefficients are *P* < 0.05.

^2^ $R_{m}^{2}$ = *R*^2^ marginal; $R_{c}^{2}$ = *R*^2^ conditional.

^3^Square root of the estimated variance associated with random effects of cow.

^4^Square root of the estimated variance associated with random effects of cow age.

^5^Square root of the estimated variance associated with random effects of year.

^6^Square root of the estimated variance associated with residual error.

## IMPLICATIONS

Results from the current study would suggest that greater cow BW will increase milk production, but it is important to note that with increasing milk production comes increased nutrient requirements. If the environment is unable to meet these increased nutrient requirements, a decrease in BCS will be observed. Dam milk production did have a positive influence on calf preweaning growth and BW. The greater BW at weaning in the offspring of dams with greater milk production, produced an advantage that was maintained throughout the feeding period to produce greater final live BW and HCW.
